# Supplementary material for: A Continuing Professional Development Program for Pharmacists Implementing Pharmacogenomics into Practice
Source: Pharmacy (Basel). 2020 Mar 28;8(2):55. doi: 10.3390/pharmacy8020055 (PMC7356265; doi:10.3390/pharmacy8020055)
Supplement: Supplementary file 1 [file pharmacy-08-00055-s001.pdf]

Table S1: Training Program Description

Session Title	Learning Objectives
**Pharmacogenomics Core Concepts**	
Pharmacogenomics: Core Concepts	1. Explain relevant genomic terminology and concepts.<br>2. Identify pharmacokinetic and pharmacodynamics sources of genetic variation.<br>3. Describe the impact of common genetic variations in CYP450 enzymes on anticipated drug response.<br>4. Identify common genetic variants in drug transporters and drug targets (e.g. SLC01B1, VKORC1) that impact response to drug therapy
Pharmacogenomics: Application to Pharmacy Practice	1. Interpret a genotype for a given P450 enzyme and describe anticipated response on drug action.<br>2. Describe the available information sources for interpreting pharmacogenomic information<br>3. Describe the role of the CPIC guidelines.<br>4. Describe the pharmacist role in the field of pharmacogenomics
Genotyping Laboratory Procedures	1. Describe the process of pharmacogenomics testing, from sample collection to report generation.
**Applications of Pharmacogenomics in Practice**	
Pharmacogenomics in Psychopharmacology: Antidepressants, Anxiolytics and Antipsychotics	1. Identify which gene-drug relationships have evidence to support their implementation for antidepressant and antipsychotic therapy.<br>2. Summarize the CPIC[1] guideline recommendations for pharmacogenetics of relevant antidepressant therapy including selective serotonin reuptake inhibitors and tricyclic antidepressants.<br>3. Discuss potential applications of pharmacogenetics to antipsychotic therapy.
Pharmacogenomics: Opioid therapy	1. Describe the impact of genetic polymorphisms on anticipated response to opioid therapy.<br>2. Describe the evidence-based guideline recommendations for codeine therapy based on CYP2D6 genotype.
Cardiovascular Pharmacogenomics	1. Describe the evidence and guideline recommendations for genotype guided warfarin dosing.<br>2. Describe the impact of CYP2C19 polymorphisms on clopidogrel therapy.<br>3. Discuss the impact of SC01B1 polymorphisms on risk of adverse effects with simvastatin therapy.
Case Discussions: Applying Pharmacogeno-mic Testing in Practice	1. Intepret a pharmacogenetic testing report.<br>2. For a given patient scenario, identify relevant pharmacogenetic variations that may impact drug therapy, and develop recommendations to optimize therapy.
Implementing Pharmacogenomic Testing into Practice: Communicating with Pati -ents and the Health Care Team	1. Describe an approach to educate both a patient and other healthcare professionals about pharmacogenomics test results.<br>2. Describe the key pieces of information to include in documentation of pharmacogenomic testing results and treatment plans.<br>3. Discuss ethical and legal implications of pharmacogenomic testing.

[1]CPIC - Clinical Pharmacogenetic Implementation Consortium https://cpicpgx.org/guidelines/
